# 43 The Development of the Preschool Life Impact Burn Recovery Evaluation Short Forms

**DOI:** 10.1093/jbcr/irae036.043

**Published:** 2024-04-17

**Authors:** Madeleine McGwin, Pengsheng Ni, Khushbu F Patel, Kate Surette, Alexandra R Gladstone, Tina L Palmieri, Michael Murphy, Frederick J Stoddard, Jr., Jeffrey C Schneider, Lewis E Kazis, Colleen M Ryan

**Affiliations:** Shriners Children's Boston, Boston, Massachusetts; Boston University School of Public Health, Boston, MA; Shriners Hospitals for Children - Boston, Allston, Massachusetts; Tulane University, New Orleans, Louisiana; Shriners' Childrens Boston, Swampscott, Massachusetts; UC Davis, Shriners Children's Northern California, Sacramento, CA; Massachusetts General Hospital/Harvard Medical School, Acton, Massachusetts; Harvard Medical School, Boston, Massachusetts; Spaulding Rehabilitation Hospital/Harvard Medical School, Boston, MA; Department of Health Law, Policy and Management, Boston University School of Public Health, Boston, MA; Massachusetts General Hospital/Shriners Children's, Boston, MA; Shriners Children's Boston, Boston, Massachusetts; Boston University School of Public Health, Boston, MA; Shriners Hospitals for Children - Boston, Allston, Massachusetts; Tulane University, New Orleans, Louisiana; Shriners' Childrens Boston, Swampscott, Massachusetts; UC Davis, Shriners Children's Northern California, Sacramento, CA; Massachusetts General Hospital/Harvard Medical School, Acton, Massachusetts; Harvard Medical School, Boston, Massachusetts; Spaulding Rehabilitation Hospital/Harvard Medical School, Boston, MA; Department of Health Law, Policy and Management, Boston University School of Public Health, Boston, MA; Massachusetts General Hospital/Shriners Children's, Boston, MA; Shriners Children's Boston, Boston, Massachusetts; Boston University School of Public Health, Boston, MA; Shriners Hospitals for Children - Boston, Allston, Massachusetts; Tulane University, New Orleans, Louisiana; Shriners' Childrens Boston, Swampscott, Massachusetts; UC Davis, Shriners Children's Northern California, Sacramento, CA; Massachusetts General Hospital/Harvard Medical School, Acton, Massachusetts; Harvard Medical School, Boston, Massachusetts; Spaulding Rehabilitation Hospital/Harvard Medical School, Boston, MA; Department of Health Law, Policy and Management, Boston University School of Public Health, Boston, MA; Massachusetts General Hospital/Shriners Children's, Boston, MA; Shriners Children's Boston, Boston, Massachusetts; Boston University School of Public Health, Boston, MA; Shriners Hospitals for Children - Boston, Allston, Massachusetts; Tulane University, New Orleans, Louisiana; Shriners' Childrens Boston, Swampscott, Massachusetts; UC Davis, Shriners Children's Northern California, Sacramento, CA; Massachusetts General Hospital/Harvard Medical School, Acton, Massachusetts; Harvard Medical School, Boston, Massachusetts; Spaulding Rehabilitation Hospital/Harvard Medical School, Boston, MA; Department of Health Law, Policy and Management, Boston University School of Public Health, Boston, MA; Massachusetts General Hospital/Shriners Children's, Boston, MA; Shriners Children's Boston, Boston, Massachusetts; Boston University School of Public Health, Boston, MA; Shriners Hospitals for Children - Boston, Allston, Massachusetts; Tulane University, New Orleans, Louisiana; Shriners' Childrens Boston, Swampscott, Massachusetts; UC Davis, Shriners Children's Northern California, Sacramento, CA; Massachusetts General Hospital/Harvard Medical School, Acton, Massachusetts; Harvard Medical School, Boston, Massachusetts; Spaulding Rehabilitation Hospital/Harvard Medical School, Boston, MA; Department of Health Law, Policy and Management, Boston University School of Public Health, Boston, MA; Massachusetts General Hospital/Shriners Children's, Boston, MA; Shriners Children's Boston, Boston, Massachusetts; Boston University School of Public Health, Boston, MA; Shriners Hospitals for Children - Boston, Allston, Massachusetts; Tulane University, New Orleans, Louisiana; Shriners' Childrens Boston, Swampscott, Massachusetts; UC Davis, Shriners Children's Northern California, Sacramento, CA; Massachusetts General Hospital/Harvard Medical School, Acton, Massachusetts; Harvard Medical School, Boston, Massachusetts; Spaulding Rehabilitation Hospital/Harvard Medical School, Boston, MA; Department of Health Law, Policy and Management, Boston University School of Public Health, Boston, MA; Massachusetts General Hospital/Shriners Children's, Boston, MA; Shriners Children's Boston, Boston, Massachusetts; Boston University School of Public Health, Boston, MA; Shriners Hospitals for Children - Boston, Allston, Massachusetts; Tulane University, New Orleans, Louisiana; Shriners' Childrens Boston, Swampscott, Massachusetts; UC Davis, Shriners Children's Northern California, Sacramento, CA; Massachusetts General Hospital/Harvard Medical School, Acton, Massachusetts; Harvard Medical School, Boston, Massachusetts; Spaulding Rehabilitation Hospital/Harvard Medical School, Boston, MA; Department of Health Law, Policy and Management, Boston University School of Public Health, Boston, MA; Massachusetts General Hospital/Shriners Children's, Boston, MA; Shriners Children's Boston, Boston, Massachusetts; Boston University School of Public Health, Boston, MA; Shriners Hospitals for Children - Boston, Allston, Massachusetts; Tulane University, New Orleans, Louisiana; Shriners' Childrens Boston, Swampscott, Massachusetts; UC Davis, Shriners Children's Northern California, Sacramento, CA; Massachusetts General Hospital/Harvard Medical School, Acton, Massachusetts; Harvard Medical School, Boston, Massachusetts; Spaulding Rehabilitation Hospital/Harvard Medical School, Boston, MA; Department of Health Law, Policy and Management, Boston University School of Public Health, Boston, MA; Massachusetts General Hospital/Shriners Children's, Boston, MA; Shriners Children's Boston, Boston, Massachusetts; Boston University School of Public Health, Boston, MA; Shriners Hospitals for Children - Boston, Allston, Massachusetts; Tulane University, New Orleans, Louisiana; Shriners' Childrens Boston, Swampscott, Massachusetts; UC Davis, Shriners Children's Northern California, Sacramento, CA; Massachusetts General Hospital/Harvard Medical School, Acton, Massachusetts; Harvard Medical School, Boston, Massachusetts; Spaulding Rehabilitation Hospital/Harvard Medical School, Boston, MA; Department of Health Law, Policy and Management, Boston University School of Public Health, Boston, MA; Massachusetts General Hospital/Shriners Children's, Boston, MA; Shriners Children's Boston, Boston, Massachusetts; Boston University School of Public Health, Boston, MA; Shriners Hospitals for Children - Boston, Allston, Massachusetts; Tulane University, New Orleans, Louisiana; Shriners' Childrens Boston, Swampscott, Massachusetts; UC Davis, Shriners Children's Northern California, Sacramento, CA; Massachusetts General Hospital/Harvard Medical School, Acton, Massachusetts; Harvard Medical School, Boston, Massachusetts; Spaulding Rehabilitation Hospital/Harvard Medical School, Boston, MA; Department of Health Law, Policy and Management, Boston University School of Public Health, Boston, MA; Massachusetts General Hospital/Shriners Children's, Boston, MA; Shriners Children's Boston, Boston, Massachusetts; Boston University School of Public Health, Boston, MA; Shriners Hospitals for Children - Boston, Allston, Massachusetts; Tulane University, New Orleans, Louisiana; Shriners' Childrens Boston, Swampscott, Massachusetts; UC Davis, Shriners Children's Northern California, Sacramento, CA; Massachusetts General Hospital/Harvard Medical School, Acton, Massachusetts; Harvard Medical School, Boston, Massachusetts; Spaulding Rehabilitation Hospital/Harvard Medical School, Boston, MA; Department of Health Law, Policy and Management, Boston University School of Public Health, Boston, MA; Massachusetts General Hospital/Shriners Children's, Boston, MA

## Abstract

**Introduction:**

The Preschool Life Impact Burn Recovery Evaluation Computer adaptive test (PS-LIBRE1-5 CAT) has been developed using item response theory methods and CAT technologies. While the CAT can provide precise, real-time assessment, it can be limited in its use due to advanced technological requirements. Therefore, the aim of the present study was to develop PS-LIBRE1-5 short forms (SFs). This is the next logical step for the PS-LIBRE1-5 project as it will improve the accessibility of the instrument in the absence of advanced computer technology.

**Methods:**

Data from the field-testing and calibration of the PS-LIBRE1-5 was assessed for SF usage. The items were iteratively added into a short form. The candidate item was selected based on maximizing the expected value of the test information function (TIF), which is the sum of TIF weighted by the sample score distribution. This process considers both information function and sample distribution, however it does not prevent from selecting an item with very high information value, covering a narrow score range. Therefore, the highest test information value for each candidate short form was constrained to a reliability value of 0.8. Differential item functioning (DIF) was examined for age, burn size, sex, parent education level, and burn location. Cronbach alpha and score correlations were used to assess internal consistency, reliability and validity. The number of items for a SF was set at 10.

**Results:**

The PS-LIBRE1-5 SFs were developed using data from 498 parents of burn survivors, ages 1 to 5 years, who participated in the field-testing of the item pool. The mean ± SD child age was 3.0 ± 1.4 years with 55.2% males. Mean TBSA burned was 4.2% and average time since burn was 1.1 years. 10-item short forms were developed to cover three PS-LIBRE1-5 scales: Communication and Language, Physical Functioning and Emotional Well-being. The DIF analysis resulted in two short forms for Communication and Language; >3 and ≤3 years old. Ceiling effects were ≤15% and floor effects were < 1% for all scales. The marginal reliability of the SFs ranged from 0.74 to 0.90. The Cronbach’s alpha ranged from 0.86 to 0.90. Score correlation of the SFs to full item bank was 0.97 to 0.98.

**Conclusions:**

The scales of the PS-LIBRE1-5 performed credibly well and were psychometrically sound in the development of SFs. Following final validation, these short forms will be tested for use in clinical practice and for future observational studies and clinical interventions.

**Applicability of Research to Practice:**

PS-LIBRE1-5 SFs have been developed and can be used to generate reasonable estimations of the CAT score for the PS- LIBRE1-5, with the goal to improve accessibility of the instrument in clinical and research settings.

